# GOLD-Induced Cytokine (GOLDIC): A Critical Review of Its Properties, Synthesis, and Biomedical Applications

**DOI:** 10.7759/cureus.52130

**Published:** 2024-01-11

**Authors:** Sankalp Yadav, Gautam Rawal, Naveen Jeyaraman, Madhan Jeyaraman

**Affiliations:** 1 Medicine, Shri Madan Lal Khurana Chest Clinic, New Delhi, IND; 2 Respiratory Medical Critical Care, Max Super Speciality Hospital, New Delhi, IND; 3 Orthopaedics, ACS Medical College and Hospital, Dr MGR Educational and Research Institute, Chennai, IND

**Keywords:** nf-κb signaling, immunomodulation, inflammatory cytokines, goldic, gold compounds

## Abstract

This narrative review examines the intricate landscape of gold-induced cytokine responses, delving into the anti-inflammatory and immune-modulating properties of gold compounds, with a primary focus on their application in treating rheumatoid arthritis and other autoimmune conditions. Utilizing a comprehensive search strategy across major scientific databases, we identified and analyzed a diverse range of studies published within the last two decades. The aim of this review is to provide a nuanced understanding of the current state of knowledge, addressing key questions regarding the mechanisms by which gold compounds modulate cytokine responses and their clinical implications. Our review encompasses an in-depth exploration of the anti-inflammatory effects of gold compounds, emphasizing their impact on pro-inflammatory cytokines such as tumor necrosis factor-alpha (TNF-α), interleukin 1β (IL-1β), and interleukin 6 (IL-6). Moreover, we investigate the lesser-explored terrain of immune modulation, shedding light on the ability of gold compounds to influence anti-inflammatory cytokines, notably interleukin 10 (IL-10). Through an extensive analysis of the literature, we unravel the multifaceted mechanisms underlying gold-induced cytokine responses, including the inhibition of nuclear factor kappa B (NF-κB) signaling and interference with janus kinase/signal transducers and activators of transcription (JAK/STAT) pathways. In exploring the clinical applications of gold-induced cytokine modulation, we synthesize findings from relevant studies, elucidating the potential of gold compounds as therapeutic agents. However, challenges such as variability in formulations and diverse cytokine assessment methods are discussed, emphasizing the need for standardization in both research and clinical settings. Looking ahead, our scoping review identifies key unanswered questions and proposes future directions for research in this domain. We discuss emerging therapeutic strategies, considering the integration of gold compounds with other modalities to optimize treatment outcomes. This comprehensive review serves as a foundational resource for researchers, clinicians, and policymakers seeking a nuanced understanding of gold-induced cytokine responses, paving the way for further advancements in this critical area of study.

## Introduction and background

The use of gold compounds in medical applications has a rich history, particularly in the context of addressing inflammatory conditions. These compounds, exemplified by aurothiomalate and auranofin, have long been recognized for their potent anti-inflammatory properties [1]. Historically, gold compounds gained prominence as a therapeutic intervention for conditions such as rheumatoid arthritis (RA) and other autoimmune diseases [2,3]. The evolution of their application over time reflects a complex interplay between scientific understanding, clinical observations, and the ongoing pursuit of effective anti-inflammatory strategies.

Background

Gold compounds, notably aurothiomalate and auranofin, have been integral components of medical arsenals aimed at combating inflammatory diseases. Originating from empirical observations, their incorporation into medical practice can be traced back to early in the 20th century. The initial recognition of gold's anti-inflammatory potential occurred serendipitously, laying the foundation for subsequent investigations into their mechanistic actions [4-6]. With a specific focus on rheumatoid arthritis, gold compounds emerged as pioneering agents in the treatment of this debilitating autoimmune condition.

The anti-inflammatory properties of gold compounds manifest through their ability to modulate cytokine responses. This complex interaction involves the intricate regulation of pro-inflammatory cytokines such as tumor necrosis factor-alpha (TNF-α), interleukin 1β (IL-1β), and interleukin 6 (IL-6) [7-10]. Understanding the nuances of gold-induced cytokine responses is paramount for unraveling the full therapeutic potential of these compounds.

Rationale for the review

In light of the historical use of gold compounds and their contemporary relevance in treating inflammatory conditions, there exists a pressing need to comprehensively understand the underlying mechanisms of gold-induced cytokine responses. The rationale for this scoping review is twofold: First, a thorough exploration of the intricate relationships between gold compounds and cytokine modulation is crucial for advancing our scientific understanding. Second, the clinical relevance of such modulation has direct implications for therapeutic strategies, especially in the context of autoimmune diseases, where dysregulated cytokine responses play a pivotal role.

As inflammatory conditions continue to pose significant challenges in healthcare, the potential therapeutic implications of gold-induced cytokine responses underscore the importance of a nuanced and updated synthesis of existing knowledge. This review aims to bridge existing gaps in our understanding, providing a comprehensive resource for researchers, clinicians, and policymakers grappling with the complexities of anti-inflammatory interventions involving gold compounds.

Objectives

The primary objectives of this scoping review are tabulated in Table 1.

**Table 1:** The primary objectives of this scoping review

Objectives
To systematically examine existing literature on gold-induced cytokine responses, focusing on the modulation of both pro-inflammatory and anti-inflammatory cytokines.
To elucidate the molecular mechanisms by which gold compounds influence cytokine expression, with a specific emphasis on the inhibition of pro-inflammatory pathways and enhancement of anti-inflammatory pathways.
To assess the clinical applications of gold-induced cytokine modulation, summarizing findings from relevant studies and highlighting areas of consensus and divergence.
To identify gaps in the current understanding of gold-induced cytokine responses and propose avenues for future research.

Research questions guiding the review

The research questions guiding the review are tabulated in Table 2.

**Table 2:** Research questions

Research questions guiding the review
How do gold compounds modulate pro-inflammatory cytokines, and what are the underlying molecular mechanisms?
In what ways do gold compounds influence anti-inflammatory cytokines, and how does this contribute to immune modulation?
What is the current state of the evidence regarding the clinical applications of gold-induced cytokine responses?
What are the remaining knowledge gaps, and what future research directions should be pursued in this field?

## Review

Gold compounds and cytokine modulation

Overview of Gold Compounds

Gold compounds, such as aurothiomalate and auranofin, have a well-established history in the treatment of inflammatory conditions, particularly rheumatoid arthritis [11]. Aurothiomalate, a gold salt, and auranofin, an oral gold compound, are among the most commonly utilized agents. These compounds exhibit distinct chemical properties and mechanisms of action, contributing to their anti-inflammatory effects [12-14].

Aurothiomalate, chemically represented as Au(TiC4H9)2OH, is administered parenterally and undergoes intracellular conversion to active forms, which may interfere with various intracellular processes [15]. Auranofin, on the other hand, is an orally administered gold compound ([(Ph3P)2Au]Cl) that undergoes bioactivation within cells, contributing to its anti-inflammatory properties [16,17].

The mechanisms of action of gold compounds are multifaceted. While the exact pathways are not fully elucidated, they are thought to involve interference with signal transduction pathways, particularly those related to inflammation. Both aurothiomalate and auranofin have been implicated in the inhibition of nuclear factor-kappa B (NF-κB) signaling, a central pathway regulating pro-inflammatory gene expression [18-20].

Anti-Inflammatory Effects

Gold compounds have demonstrated significant anti-inflammatory effects by modulating the expression of pro-inflammatory cytokines. Studies have consistently reported the inhibition of key cytokines such as TNF-α, IL-1β, and IL-6 in response to gold treatment [21,22]. These cytokines play pivotal roles in the initiation and propagation of inflammatory responses, and their dysregulation is a hallmark of various autoimmune diseases.

In experimental models, aurothiomalate has been shown to effectively suppress TNF-α production in synovial fibroblasts derived from rheumatoid arthritis patients [23,24]. Auranofin has demonstrated inhibitory effects on IL-1β and IL-6 in models of inflammatory arthritis, underscoring its potential as a modulator of diverse pro-inflammatory pathways [25].

While the anti-inflammatory effects are consistent, variations in responses have been observed in different experimental models. Factors such as the specific cell type, disease model, and gold compound formulation may contribute to these variations. Understanding the context-dependent nature of these responses is crucial for optimizing the therapeutic application of gold compounds.

Immune Modulation

Beyond their inhibitory effects on pro-inflammatory cytokines, gold compounds have shown promise in immune modulation by influencing anti-inflammatory cytokines, particularly IL-10 [26,27]. IL-10 is a key regulator of immune responses, exerting anti-inflammatory effects by suppressing the production of pro-inflammatory cytokines [28,29].

Studies have indicated that aurothiomalate treatment enhances the expression of IL-10 in activated macrophages [30,31]. This dual action of inhibiting pro-inflammatory cytokines while promoting anti-inflammatory cytokines suggests a potential for gold compounds to balance immune responses.

The impact of gold-induced immune modulation on immune response balance is of particular significance in the context of autoimmune diseases, where dysregulated immune responses contribute to pathology. A nuanced understanding of these immune-modulating properties is essential for harnessing the full therapeutic potential of gold compounds in conditions characterized by immune system dysregulation.

Mechanisms of action

NF-κB Signaling

Gold compounds, such as aurothiomalate and auranofin, have been implicated in the modulation of key intracellular signaling pathways, particularly the NF-κB pathway [20]. NF-κB is a crucial transcription factor involved in the regulation of genes associated with inflammation, immune responses, and cell survival [32-34].

Several studies suggest that gold compounds exert inhibitory effects on NF-κB signaling, leading to downstream alterations in cytokine regulation. In an experimental model, auranofin was shown to inhibit NF-κB activation in synoviocytes derived from rheumatoid arthritis patients, resulting in decreased production of pro-inflammatory cytokines such as TNF-α [35,36]. Aurothiomalate has similarly demonstrated the ability to suppress NF-κB activation, thereby modulating the expression of inflammatory mediators [37,38].

**Figure 1:**
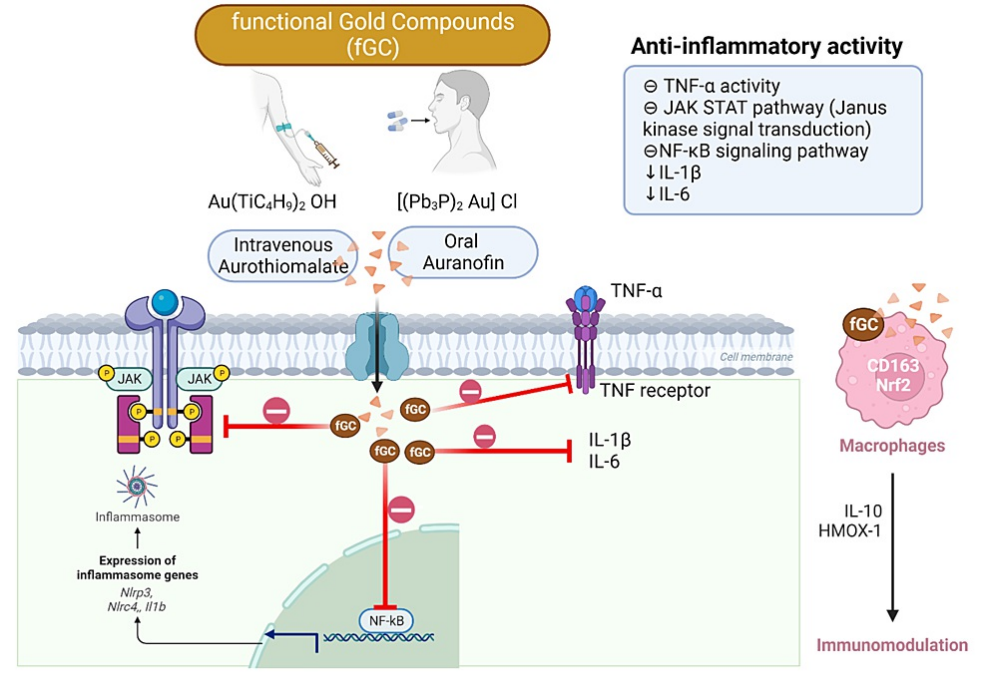
Mechanisms of action of gold compounds IL: Interleukin NF-κB: Nuclear factor-kappa B TNF-α: Tumor necrosis factor-alpha Picture courtesy Dr. Sankalp Yadav

The inhibition of NF-κB signaling by gold compounds holds significant implications for cytokine regulation. NF-κB governs the expression of various pro-inflammatory cytokines, including IL-1β and IL-6 [39,40]. By interfering with NF-κB activation, gold compounds have the potential to downregulate the production of these cytokines, contributing to their anti-inflammatory effects. This modulation of NF-κB signaling represents a key mechanistic insight into the anti-inflammatory properties of gold compounds Figure 1.

JAK/STAT Pathways

In addition to NF-κB signaling, emerging evidence suggests that gold compounds interfere with the Janus kinase/signal transducer and activator of transcription (JAK/STAT) pathways [41]. The JAK/STAT pathway is integral to cellular responses to various cytokines and growth factors, playing a crucial role in immune regulation and inflammation [42,43].

Gold-induced interference with JAK/STAT pathways has been observed in studies investigating the effects of aurothiomalate and auranofin. Auranofin, for instance, has been reported to inhibit JAK1/3 phosphorylation in activated T cells, influencing downstream STAT activation [25]. Aurothiomalate has demonstrated similar interference with JAK/STAT signaling in models of inflammatory arthritis [44,45].

The downstream effects of gold-induced interference with JAK/STAT pathways extend to cytokine expression [44]. JAK/STAT signaling is intricately involved in the regulation of cytokines such as interferons and interleukins [46]. By modulating these pathways, gold compounds have the potential to influence the production and balance of various cytokines, thereby shaping the immune response.

Understanding the dual impact of gold compounds on both NF-κB signaling and JAK/STAT pathways provides a comprehensive view of their mechanisms of action. These interactions contribute to the modulation of cytokine profiles, representing key molecular events that underpin the therapeutic effects of gold compounds in the context of inflammatory and autoimmune conditions.

Clinical applications and challenges

Clinical Studies

Clinical studies exploring the effects of gold compounds on cytokine responses have provided valuable insights into their potential therapeutic applications. A number of investigations have focused on conditions such as RA and other autoimmune disorders.

Findings from clinical studies suggest that gold compounds exert their anti-inflammatory effects, at least in part, by downregulating pro-inflammatory cytokines. Reductions in circulating levels of TNF-α, IL-1β, and IL-6 have been observed in patients receiving gold-based therapies [47,48]. These changes are often associated with improvements in clinical symptoms and disease progression.

The clinical relevance of gold-induced cytokine modulation extends beyond RA, with studies exploring their potential in other inflammatory conditions. Challenges, however, arise concerning the variability in patient responses and the need for personalized treatment strategies. Despite these challenges, the accumulated evidence from clinical studies positions gold compounds as viable options for managing cytokine-driven inflammatory diseases.

Variability in Formulations

A significant challenge in the clinical application of gold compounds lies in the variability of formulations. Different gold salts, such as aurothiomalate and auranofin, exhibit distinct pharmacokinetic and pharmacodynamic properties. This variability poses challenges for standardizing treatment regimens and comparing outcomes across studies.

The variability in formulations extends to differences in dosing schedules, routes of administration, and bioavailability. These variations can influence the magnitude of cytokine modulation and, consequently, the therapeutic outcomes. For example, auranofin, an oral gold compound, may have different effects on cytokine responses compared to injectable gold salts.

Standardization of gold compound formulations is crucial for ensuring consistency in research and clinical practice. Addressing this challenge involves establishing clear guidelines for dosing, administration, and monitoring. Collaborative efforts between researchers, clinicians, and pharmaceutical manufacturers are essential to establishing standardized protocols that optimize the therapeutic potential of gold compounds.

Assessment Methods

The diversity in cytokine assessment methods across different studies introduces another layer of complexity in interpreting results and comparing outcomes. Variations in sample collection, assay techniques, and detection limits can influence the reported cytokine levels.

Some studies employ enzyme-linked immunosorbent assays (ELISAs) to quantify cytokines, while others utilize multiplex immunoassays or gene expression profiling. These differences in assessment methods may contribute to discrepancies in reported cytokine levels and hinder the ability to draw consistent conclusions.

Exploring variations in assessment methods is crucial for understanding the nuances of gold-induced cytokine responses. Efforts toward standardization in cytokine measurement techniques are essential to enhance result reproducibility and comparability across studies. Consensus on optimal assessment methods, including validation studies, can contribute to a more cohesive understanding of the immunomodulatory effects of gold compounds.

Future directions

Unanswered Questions

While significant progress has been made in unraveling the complex interplay between gold compounds and cytokine responses, several unanswered questions persist, pointing towards avenues for future research:

Mechanistic elucidation: Despite advancements, the precise molecular mechanisms governing gold-induced cytokine modulation remain incompletely understood. Further research is warranted to elucidate the intricate signaling pathways and cellular targets involved.

Cell-type specificity: The differential effects of gold compounds on various cell types and immune cells remain a subject of exploration. Understanding cell-type-specific responses is crucial for tailoring interventions to specific diseases and optimizing therapeutic outcomes.

Long-term effects: The long-term impact of gold-based treatments on cytokine regulation and immune function is a critical area requiring attention. Longitudinal studies assessing sustained efficacy and potential adverse effects over extended treatment durations are essential.

Patient stratification: Variability in patient responses to gold compounds raises the question of patient stratification. Identifying biomarkers or genetic factors that predict individual responses can aid in personalizing treatment strategies for enhanced efficacy.

Combination therapies: Exploring the synergistic effects of gold compounds in combination with other therapeutic agents is an area of growing interest. Investigating potential interactions and optimizing combination regimens may offer enhanced therapeutic benefits.

Emerging therapeutic strategies

Optimizing Gold-Based Treatments

Nanoformulations: Developing nanoscale formulations of gold compounds may enhance their bioavailability and therapeutic efficacy. Nanoformulations can improve drug delivery by targeting specific cells or tissues, potentially reducing side effects, and optimizing cytokine modulation [49-51].

Targeted delivery systems: Advancements in targeted drug delivery systems allow for the specific delivery of gold compounds to inflamed tissues. This approach minimizes systemic exposure, reducing the potential for off-target effects while maximizing therapeutic impact [52,53].

Integration With Other Therapeutic Modalities

Biological therapies: Combining gold compounds with biological therapies, such as monoclonal antibodies targeting specific cytokines or immune cells, presents a promising avenue. Synergistic effects may be achieved by simultaneously targeting multiple points in the inflammatory cascade [54].

Precision medicine approaches: Integrating gold-based treatments into precision medicine frameworks holds potential. Tailoring interventions based on individual patient characteristics, including genetic, immunologic, and cytokine profiles, may optimize treatment outcomes [55,56].

Immunomodulatory therapies: Exploring the integration of gold compounds with other immunomodulatory agents, such as small molecules or cell-based therapies, offers a multifaceted approach to immune regulation. Combinatorial strategies may provide enhanced efficacy and reduced toxicity [27].

In conclusion, this scoping review has provided a comprehensive exploration of the intricate relationship between gold compounds and cytokine responses, with a specific focus on their anti-inflammatory properties. The historical significance of gold compounds in medicine, particularly in the treatment of conditions like rheumatoid arthritis, sets the stage for understanding their evolving role in modern therapeutic strategies.

The review has highlighted the multifaceted mechanisms through which gold compounds, such as aurothiomalate and auranofin, modulate cytokine expression. By inhibiting key signaling pathways like NF-κB and interfering with JAK/STAT pathways, these compounds exhibit a nuanced ability to downregulate pro-inflammatory cytokines while also influencing anti-inflammatory mediators. The clinical applications of gold compounds, as evidenced by studies in conditions like rheumatoid arthritis, underscore their potential as valuable immunomodulatory agents.

However, the field is not without challenges. Variability in formulations, both in terms of different gold compounds and diverse administration methods, poses challenges for standardization in research and clinical practice. The review also emphasizes the impact of variations in assessment methods on result interpretation and comparability, urging the need for standardized approaches in cytokine measurement.

Looking to the future, numerous unanswered questions beckon further research. Mechanistic elucidation, cell-type specificity, and the long-term effects of gold-based treatments are key areas requiring in-depth exploration. Patient stratification based on individual responses and the integration of gold compounds with emerging therapeutic strategies, such as nanoformulations and precision medicine approaches, present exciting avenues for optimizing treatment outcomes.

## Conclusions

In conclusion, the future of gold-induced cytokine modulation lies in addressing existing knowledge gaps, refining treatment strategies based on patient characteristics, and exploring innovative approaches for enhanced therapeutic outcomes. To summarize, while the anti-inflammatory potential of gold compounds in modulating cytokine responses is evident, continued research and collaborative efforts are essential to harnessing their full therapeutic benefits. The dynamic landscape of immunomodulation, coupled with advancements in drug delivery and personalized medicine, holds promise for shaping the future of gold-induced cytokine modulation as a cornerstone in managing inflammatory and autoimmune diseases.
